# Editorial: Yoga for pain relief

**DOI:** 10.3389/fpain.2024.1422483

**Published:** 2024-05-06

**Authors:** Pradeep M. K. Nair, Jyoti Keswani, H. S. Vadiraja

**Affiliations:** ^1^ Department of Integrative Oncology, Mirakle Integrated Health Centre, Pollachi, India; ^2^ Department of Yoga, Sant Hirdaram Medical College for Naturopathy and Yogic Sciences for Women, Bhopal, India; ^3^ Department of Naturopathy, Central Council for Research in Yoga and Naturopathy, New Delhi, India

**Keywords:** yoga, pain, complementary and alternative medicine, mind—body approaches, yoga therapy

**Editorial on the Research Topic** Yoga for pain relief

Over the past three decades, yoga has evolved as a potential psychosomatic tool, leaving behind its past image as a mere spiritual tool. While the evidence suggesting the positive role of yoga in the management of non-communicable diseases is overwhelming, only limited research has been conducted demonstrating the role of yoga in pain relief. Unlike other traditional approaches, which are primarily focused on physiological wellbeing, yoga has a dualistic approach that can influence both the body and the mind. Pain, a normal physiological phenomenon is a common symptom arising from the underlying inflammatory process in the body. However, there is huge inter-individual variability in the perception of pain. The inter-individual variability in the experience of pain is attributed to multiple genetic and psychosocial factors (1). This make pain as a very individualistic symptom, that requires interventions that can strengthen both physical and psychological attributes. Therefore, utilizing yoga as a personalized psychosomatic tool in the management of pain will have a meaningful impact on the patients, as it can modify their individual perceptions and thresholds.

This research topic on yoga for pain relief has provided different perspectives on the utility of yoga in pain. Chopra et al., have presented a complex adaptive system within our body from a yogic perspective, highlighting the importance of integrating yoga into pain management strategies. The evidence emerging from this study compels the use of yoga as a holistic tool in pain as opposed to the discrete reductionist approach. This research topic also presents an overview of the sociodemographic features of patients who took yoga for pain in India. Nair et al., in this retrospective study have shown musculoskeletal pain to top the list of yoga seekers, and the majority of the participants who seek yoga for pain relief were female. Additionally, it was observed that, in India, yoga is mostly used as an integrative therapy with other Ayush disciplines, especially Naturopathy. The authors reported that the majority of the participants who took yoga were not covered under any insurance programs. This indicates the need for policy-level interventions to increase the utilization of yoga by the general public, despite its increasing popularity.

This research topic also demonstrates the medical use of yoga therapy in conventional pain management settings. Arya et al., in their study involving 108 participants, have shown that yoga not only reduces pain but also improves the quality of life and reduces stress levels among patients with chronic low back pain. Additionally, this study reported an increase in the nociceptive flexion-reflex threshold among medical yoga therapy participants. This indicates that yoga therapy can modulate the neurophysiological correlates related to pain, resulting in increased pain tolerance capacity. Kanchibhotla et al., in their review, highlighted the role of yoga in altering the stress and hormonal pathways in patients with dysmenorrhoea. Yoga practices like asana, pranayama, and yoga nidra were identified as the most beneficial practices for relieving dysmenorrhoea-related pain. The alteration in the pain symptoms in this cohort can be attributed to the relaxation response induced by yoga in the neuroendocrine system, which impacts both pain and its perception.

Yoga is a complex mind-body approach, so is the pain sensation. Pain equally impacts the physical, emotional, social, and spiritual sheaths of the patient. Yoga is further referred to as “the discipline which severs the connection with that which causes suffering” in Bhagavat Gita VI 23, an ancient scripture from India. Considering the physical and psychological intricacies associated with pain, yoga emerges as a comprehensive, evidence-based approach that can be readily integrated into pain management strategies. In conclusion, this research topic offers a 360-degree view of utilising yoga in pain settings. This topic provides deep insights on multiple facets of yoga, such as unravelling the underlying mechanisms for employing yoga in pain, the clinical utility of yoga, the prevalence of yoga practice in pain, and the policy recommendations. As yoga continues to evolve as a promising therapeutic tool, the knowledge gained through this topic may contribute significantly in upregulating its growth as a scientific remedy for pain.
